# Supplementary material for: An OMA1 redox site controls mitochondrial homeostasis, sarcoma growth, and immunogenicity
Source: Life Sci Alliance. 2023 Apr 5;6(6):e202201767. doi: 10.26508/lsa.202201767 (PMC10078952; doi:10.26508/lsa.202201767)
Supplement: Supplementary file 5 [file LSA-2022-01767_Supplemental_Data_1.docx]

**REAGENTS AND TOOLS TABLE**

| Reagent/Resource | Reference or Source | Identifier or Catalog Number |
| --- | --- | --- |
| Experimental Models |  |  |
| C57BL/6J (*M. musculus*) | Janvier Laboratories | C57BL/6J |
| NMRI Nude (*M. musculus*) | Janvier Laboratories | Rj:NMRI-Foxn1nu/nu |
| XCR1-iCre-mTFP1 x Rosa DTA | Bernard Malissen’s lab | (Wohn *et al*, 2020) |
| MCA205 OMA1 WT |  | Kindly provided by Yann KERDILES  (CIML- Marseille) |
| MCA205 OMA1 CTRL | This study |  |
| MCA205 OMA1 WT/C403A | This study |  |
| MCA205 OMA1 C403A | This study |  |
| Recombinant DNA |  |  |
| pCMV-PE2 | Addgene | 132775 |
| pU6-pegRNA-GG-acceptor | Addgene | 132777 |
| pLKO.1-puro GFP |  | (Phelan *et al*, 2018) |
| peGFP-C1 vector plasmid | Clontech |  |
| Antibodies |  |  |
| Mouse monoclonal antibody OMA-1 (1:100) | Santa Cruz - H-11 | sc-515788 |
| Rabbit monoclonal antibody OPA-1 (1:1000) | Cell Signalling - D6U6N | #80471 |
| Mouse monoclonal antibody DELE1 (1:100) | Santa Cruz –D-11 | sc-515080 |
| Rabbit monoclonal antibody TOMM (1:1000 WB – 1:500 IF) | Abcam - EPR15581-54 | ab186735 |
| Mouse Actine  (1:1000) | Sigma-Aldrich- AC40 | A4700-100UL |
| Purified Rat CD16-CD32 | BD Pharmingen – 2 4G2 | 553141 |
| Armenian hamster monoclonal anti-CD11c BUV395 (1:200) | BD Optibuild – N418 | 744180 |
| Rat monoclonal anti-CD24 BUV737 (1:1000) | BD Horizon – M1/69 | 612832 |
| Rat monoclonal anti-Ly6C BV421 (1:300) | BD Horizon – AL-21 | 562727 |
| Rat monoclonal anti-CD11b BV510 (1:100) | BD Horizon – M1/70 | 562950 |
| Biotin Rat monoclonal anti-CD44 (1:300) | BD Pharmingen – IM7 | 553132 |
| Streptavidin BV605 (1:500) | N/A | 563260 |
| BV711 Armenian Hamster anti-TCRab (1:100) | BD Horizon – H57-597 | 563135 |
| BV786 Mouse anti-CD45.2 (1:500) | BD Horizon - 104 | 563686 |
| Rat anti-CD4 PerCP-Cy5.5 (1 :200) | BD Pharmingen – RM4-5 | 561115 |
| Rat anti-FoxP3 PE (1 :200) | Invitrogen - FJK-16s | 12-5773-82 |
| Mouse anti-NK1.1 PE-CF594 (1:100) | BD Horizon – PK136 | 562864 |
| Rat anti-CD8a PE-Cy5 (1:300) | BD Pharmingen – 53-6.7 | 553034 |
| Rat anti-CD62L PE-Cy7 (1:100) | BD Pharmingen – MEL-14 | 560516 |
| Rat anti-IA-IE AF700 (1 :500) | Biolegend – M5/114.15.2 | 107622 |
| Rat anti-Ly6G APC-Cy7 (1:300) | BD Pharmingen – 1A8 | 560600 |
| Rat anti-CD4 FITC (1:200) | BD Pharmingen – RM4-5 | 553047 |
| Mouse anti-CD64 (1:300) | Biolegend – X54-5/7.1 | 139306 |
| anti-Ly6G BUV395 | BD Biosciences – 1A8 | 565964 |
| anti-CD45.2 BUV737 | BD Biosciences – 104 | 612778 |
| anti-CD11b Pacific Blue | Biolegend – M1/70 | 101224 |
| anti-IA-IE BV711 | BD Biosciences – M5/114.15.2 | 563414 |
| anti-Ly6C BV605 | BD Biosciences – AL-21 | 563062 |
| anti-F4-80 BV785 | Biolegend – BM8 | 123141 |
| anti-CD8a PerCP Cy5.5 | BD Biosciences Pharmingen – 53.6.7 | 550765 |
| anti-CD11c PE-Cy7 | Invitrogen – PE-Cy7 | 25-0114-82 |
| anti-CD19 PE-Cy5 | Biolegend – 6D5 | 115509 |
| anti-CD4 APC-eF780 | eBiosciences – GK1.5 | 47-0042-82 |
| HRP-linked goat anti-rabbit IgG | Jackson ImmunoResearch | 111-035-144 |
| HRP-linked goat anti-mouse IgG | Sigma Aldrich | A0168 |
| Isotype CTRL Rat IgG1, κ | BioXcell- HRPN | BX-BE0088 |
| aCD8 Rat IgG1, κ | BioXcell- RMP1-14 | BX-BE0146 |
| Alexa Fluor® 594 AffiniPure Donkey Anti-Rabbit IgG (H+L) | Jackson ImmunoResearch | 711-585-152 |
| Oligonucleotides and others |  |  |
| PCR primers | This study | Appendix |
| Chemicals, Enzymes and other |  |  |
| Bsa-HFv2 | NEB | [#R3733](https://international.neb.com/products/r3733-bsai-hf-v2) |
| DPBS | Gibco - ThermoFisher Scientific | 14200-067 |
| DMEM F12 | Gibco - ThermoFisher Scientific | 11320-074 |
| RPMI Medium 1640 | Gibco - ThermoFisher Scientific | 21875-034 |
| Fetal Bovine Serum | Pan Biotech | P30-3306 |
| Sodium Pyruvate | Gibco - ThermoFisher Scientific | 11306-039 |
| L-Glutamine | Gibco - ThermoFisher Scientific | 25030-024 |
| Pen Strep | Gibco - ThermoFisher Scientific | 15140-122 |
| eBioscience 1X RBC Lysis Buffer | Invitrogen | 00-4333-57 |
| Precision Plus Protein™ Kaleidoscope™ Prestained Protein Standards | BioRad | 1610375 |
| MitoTracker Green | Invitrogen- ThermoFisher Scientific | M7514 |
| MitoTracker Deep Red FM | Invitrogen- ThermoFisher Scientific | M22426 |
| Annexin V PE | Pharmingen | 65875H |
| Live Dead Fixable Blue Dead Cell stain Kit | Invitrogen- ThermoFisher Scientific | L23105 |
| FoxP3/Transcription Factor Staining Buffer Set | Invitrogen- ThermoFisher Scientific | 00-5223-56 |
| Sphero Blank Calibration particles | BD Biosciences | 556296 |
| 0.5M EDTA pH 8.0 | Invitrogen- ThermoFisher Scientific | AM9261 |
| Mitochondrial Isolation Kit for Mammalian cells | ThermoScientific | 89874 |
| DMSO | Sigma Aldrich | D8418 |
| RNeasy Mini Kit | QIAGEN | 74104 |
| DNeasy Blood & Tissue Kit | QIAGEN | 69504 |
| CCCP | EMD Millipore Corporation | 215911 |
| TPEN | Sigma Aldrich | P4413 |
| Staurosporine | Sigma Aldrich | S5921 |
| Bortezomib | Sigma Aldrich | 5043140001 |
| Software |  |  |
| Image J 1.53 F51 | N/A | N/A |
| Diva software9.0 | BD | N/A |
| FlowJo 10.8.1 | Becton Dickinson & Company | N/A |
| Imaris 9.5.1 | Oxford instruments | N/A |
| Zen Black 2.3 SP1 FP3 v14.0.0.0 | Zeiss | N/A |
| AlphaFold | DeepMind | N/A |
| Pymol Molecular Graphic Systems. Version 2.5.4 | Schrödinger LCC | N/A |
| Steve | Nanolive | N/A |
| Eve Analytics | Nanolive | N/A |
| Other |  |  |
| NuPAGE 4-12% Bis-Tris Gel | Invitrogen | NP0335BOX |
| NativePAGE™ 3 - 12 %, Bis-Tris, 1,0 mm, minigels | Invitrogen | BN2011BX10 |
| Native Mark unstained protein standard | Invitrogen | LC0725 |
| NativePAGE 5% G-250 Sample Additive | Invitrogen | BN2004 |
| NativePAGE Running Buffer | Invitrogen | BN2001 |
| NativePAGE Cathode Buffer Additive | Invitrogen | BN2002 |
| Native PAGE Sample Buffer | Invitrogen | BN20032 |
| Imperial Protein Stain | PIERCE | 24615 |
| Annexin V Buffer | Life technologies | V13246 |
| Tumor Dissociation Kit, mouse | Miltenyi Biotec | 130-096-730 |
| CD45 Microbeads, mouse | Miltenyi Biotec | 130-052-301 |
| Pierce BCA Protein Assay Kit | ThermoFischer Scientific | 23225 |
| Amersham Hyperfilm MP | GE HealthCare Limited | 28906850 |
| LSM 880 – AiryScan Module | Zeiss | N/A |
| Fluoromount-G with DAPI | Invitrogen | 00-4959-52 |
