# Supplementary material for: An OMA1 redox site controls mitochondrial homeostasis, sarcoma growth, and immunogenicity
Source: Life Sci Alliance. 2023 Apr 5;6(6):e202201767. doi: 10.26508/lsa.202201767 (PMC10078952; doi:10.26508/lsa.202201767)
Supplement: Supplementary file 6 [file LSA-2022-01767_Supplemental_Data_2.docx]

| Primer name | Sequence 5’-3’ |
| --- | --- |
| OMA1 screening WT F | TTAACTCTGTGTTTAGGCATG |
| OMA1 screening WT R | TTAGTGGGGTTAGCTCACC |
| OMA1 screening C403A F | TTAACTCTGTGTTTAGGCAGC |
| OMA1 screening C403A R | TTAGTGGGGTTAGCTCACC |
| OMA1 WT PCR F | CATCTGCAAGGGTTCAGGCT |
| OMA1 WT PCR R | AACACTACAAAGAGCAGTCCAAA |
| OPA 1 F | TGGAAAATGGTTCGAAGTCAG |
| OPA1 R | CATTCCGTCTCTAGGTTAAAGCG |
| POLG1 F | GATGAATGGGCCTACCTTGA |
| POLG1 R | TGGGGTCCTGTTTCTACAGC |
| ND1 F | CAAACACTTATTACAACCCAAGAACA |
| ND1R | TCATATTATGGCTATGGGTCAGG |

**Supplementary Materials**
